# Measuring and understanding information storage and transfer in a simulated human gut microbiome

**DOI:** 10.1371/journal.pcbi.1012359

**Published:** 2024-09-17

**Authors:** Hannah Zoller, Carlos Garcia Perez, Javier Betel Geijo Fernández, Wolfgang zu Castell

**Affiliations:** 1 Department Geoinformation, Helmholtz Centre Potsdam – GFZ German Research Centre for Geosciences, Potsdam, Germany; 2 DIGIT Digital Transformation & IT, Helmholtz Munich, Germany; 3 Department of Microbiology and Ecosystem Science, University of Vienna, Austria; 4 Department of Mathematics, Technical University of Munich, Germany; University of California San Diego, UNITED STATES OF AMERICA

## Abstract

Considering biological systems as information processing entities and analyzing their organizational structure via information-theoretic measures has become an established approach in life sciences. We transfer this framework to a field of broad general interest, the human gut microbiome. We use BacArena, a software combining agent-based modelling and flux-balance analysis, to simulate a simplified human intestinal microbiome (SIHUMI). In a first step, we derive information theoretic measures from the simulated abundance data, and, in a second step, relate them to the metabolic processes underlying the abundance data. Our study provides further evidence on the role of active information storage as an indicator of unexpected structural change in the observed system. Besides, we show that information transfer reflects coherent behavior in the microbial community, both as a reaction to environmental changes and as a result of direct effective interaction. In this sense, purely abundance-based information theoretic measures can provide meaningful insight on metabolic interactions within bacterial communities. Furthermore, we shed light on the important however little noticed technical aspect of distinguishing immediate and delayed effects in the interpretation of local information theoretical measures.

## Author summary

The idea of considering biological systems as information processing systems has recently gained increasing attention in life sciences. Following this approach, one assumes that every agent of a living system stores and transfers information and that those operations reflect biological processes. Consequently, one should be able to gain knowledge on a system’s biological processes by measuring its information processing. An established mathematical framework to quantify information processing based on observational data is given by information theory. We explore this idea in the context of bacterial community analysis in the human gut habitat. This complex system is a common subject of ongoing research due to its impact on immunity and health. To keep complexity manageable and allow for controlled intervention, we simulate metabolic interaction of seven key species, representing the essential metabolic potential of the human gut microbiome. However, our approach can likewise be directly applied to *in vivo/in vitro* data. Estimating information storage and transfer from the resulting abundance data, we first identify a technical artefact, whose disregard can lead to strong misinterpretations of information processing. Second, making use of the metabolic data that the simulation provides, we show that information storage and transfer indeed relate to environmental conditions and species’ interactions in a meaningful way. We derive general principals for the interpretation of these measures, that allow us to infer patterns of bacterial interaction without the need to measure chemical processes. In this sense, our approach could provide an alternative way to study impacts of dietary plans or medical interventions on the community level.

## Introduction

One way to approach the question of what constitutes biological systems is to understand them as information-processing entities [1–3]. Living systems sense their environment and make use of this information to react. Next to exchange of mass and energy, transfer of information is one of the key characteristics underlying biological systems (see [1, 4]). Information theory [5] provides a framework to measure and analyze these operations in physical systems. Information processing can be understood as “computing” in the sense of transferring information, transforming, and storing it. Following this paradigm, the interaction of a set of biological agents can be considered as “computing system” (see, e.g., [2, 6]). Hereby, agents can make use of their own past states (*information storage*), or information gained from interaction with other agents (*information transfer*). Whatever information cannot be captured as storage or transfer will be subsumed as *intrinsic uncertainty* [7]. This comprises aspects of environmental conditions, randomness, or information being actively modified. Identifying the evolving degree to which agents make use of information sources, i.e. studying their *information decomposition*, can yield insights into the systems’ internal organizational structure and allows to examine interactions of living organisms even without the precise observation of biochemical processes (see, e.g. [8, 9]).

Understanding life as a “computing” system goes back to Erwin Schrödinger [4]. Various computational characteristics of living systems have been recently studied. Using information theory, Flack identifies hierarchies in living systems [8]. Similarly, Krakauer et al. discuss the information theory of individuality [9]. Lizier, Prokopenko, Zomaya and others have proposed information-theoretic measures that allow analyzing information processing in systems of interacting agents [7, 10–12]. In addition to the exemplary application to swarm algorithms [6], such analyses are primarily used in the field of neuroscience [13, 14]. In the present paper we explore the idea of using information processing as a means to infer biological interactions without assuming any further prior knowledge about the nature of these interactions.

Various measures for different aspects of information in information processing systems have been proposed (see, e.g. [15]). In particular, Schreiber’s transfer entropy [16] is used as a measure of information transfer, while active information storage [11] serves as a measure of the actively used memory of an agent. In accordance with the basic idea of information decomposition, Schreiber’s definitions are based on time series of agents’ states. Heuristically, (active) information storage of agent A is defined as the amount of uncertainty about A’s next state that can be reduced by the knowledge of its past states. Analogously, information transfer from agent B to agent A is defined as the amount of uncertainty about agent A’s next state that can be reduced by the additional knowledge of agent B’s past states. The estimation of these quantities depends on choosing a fixed number of of past states of an agent to be used for the estimation, the so-called *history length*. Intuitively, history length should exactly cover the length of the agent’s “memory”.

The exact interpretations of these quantities of information processing depend on the specific system and its environment. On a certain level of abstraction, information storage measures the independence of an agent’s development from its surrounding, while information transfer measures development driven by interaction. If an interaction between two agents A and B is being reflected in their states (we call this an *effective interaction*), a transfer of information will be measurable on the basis of two corresponding time series covering the agent’s ‘state’. It is in this sense that dynamic information decomposition captures the underlying organizational structure of a complex system.

The aim of this work is to explore the extent to which microbial communities can be understood as “computational systems”, and what insight this perspective provides on the biology of interactions both *within* a community and between a community and its environment. The latter is of particular relevance in host-microbiome interaction. An essential component of human interaction with its environment is given by the host-microbe system of the gastrointestinal tract [17–19]. However, a better understanding of this system is still a challenging research question due to the complexity and dynamics of the human microbiome. Uncovering interactions among species of the gut microbial community and examining corresponding effects on human health is subject of ongoing research [20–22]. New tools and concepts towards a better understanding of this system within its environment in a holistic sense are therefore of great interest for future studies on diagnosis and modulation. The central research claim we address in this paper therefore is the following: If indeed, microbial communities can be understood as “computing” entities, it should be possible to infer meaningful relations between the community’s information-theoretic structure and microbiome(-host) interactions. Using the purely abundance-based measures of information transfer and active information storage, this would allow us to gain insight on microbial interaction patterns *without the knowledge of metabolic processes*.

To make this hypothesis testable, we focus on a reduced representation of the intestinal microbial community, the Simplified Human Intestinal Microbiota (SIHUMI). This highly standardized community, which has been introduced by Becker et al. [23], consists of seven species, which represent the key functional capabilities of the human intestinal microbiome in both composition and fermentation capacities [24]. In our framework, we consider each species as an agent, all of which together form a joint complex system, i.e. a system of interacting agents. The SIHUMI community consists of specific strains of *Anaerostipes, Bacteroides, Bifidobacterium, Blautia, Clostridium, Escherichia*, and *Lactobacillus*. Genome sequences are available for all seven organisms, such that the metabolic potential of the community is well understood. Instead of using data from fermentation systems, we base our analysis on modeled data. We use BacArena, a software combining agent-based modelling and flux-balance analysis, to simulate the SIHUMI community [25]. Using *simulated* abundance data enables us to compare our information theoretic measures to the metabolic processes underlying the abundance data.

To keep our approach viable also for *in vitro* analysis, we need to define the state of an agents in terms of a quantity which can be measured also in *in vivo* systems. Therefore, we define the “state” of an agent at a given time in terms of the number of individuals of the respective species, i.e. its abundance at this time. The ability to realistically model microbial interactions based on metabolic modeling has been proven for BacArena. In particular, intestinal microbial communities, such as SIHUMI, have been shown to be well reflected by corresponding BacArena simulations [25]. The set-up of our *base simulation* follows the set-up for the simulation of the seven SIHUMI strains in [25]. In particular, we use the same growth stimulating initial *base medium*. In order to enforce dynamics of collapse and recovery throughout the simulation, we intervene with the system by adding the base medium several times in so-called *feeding events*.

There are several reasons why it is beneficial to base this first systematic assessment of information processing in a microbial community in *in silico* data. Primarily, it enables interventions that would be difficult to carry out in *in vitro* experiments. Nevertheless, the simulation comprises all essential interactions, such as growth and occupation of space, as well as metabolic degradation within a fully controlled set-up. In addition, the actual state of the system as a whole, including all metabolic processes, is known at all times, such that methodological artefacts can be easily separated from signals of community interaction to be studied during the analysis.

In this study, we infer signals of transfer entropy and active information storage from simulated time series of microbial abundance data and explore the potential of these quantities of information decomposition with regard to their potential for biological interpretation. Our results support the usefulness of active information storage as an indicator of sudden internal change. Concerning the biological interpretation of information transfer, our *in silico* experiments support two hypotheses:

**Hypothesis 1**. Information transfer captures coherence of the community in reaction to environmental changes.
**Hypothesis 2**. Information transfer captures coherence resulting from effective interaction among members in the community.

Note that the notion of *coherence* has appeared in similar contexts before. For example, Wang et al. [6] use it to characterize phases of high information transfer and storage in the distributed computation of swarms.

In order to test for stability, we repeat all estimations for varying history length. In doing so, we observe the occurrence of *delayed effects*. These technical artefacts are signals in information transfer/active information storage that point to past phenomena in the abundance time series rather rather than to concurrent ones. They can occur in any kind of history-based information theoretical measure and, if kept unnoticed, may lead to severe misinterpretations. We contrast them with so-called *immediate effects*, characterized by being stable with respect to changing the parameter of history length. Commonly, immediate effects are of higher interpretive relevance.

Eventually, we discuss our results in the context of general principles that Wang et al. suggest in their information-theoretical analysis of swarm dynamics [6].

## Results

**Fig 1:**
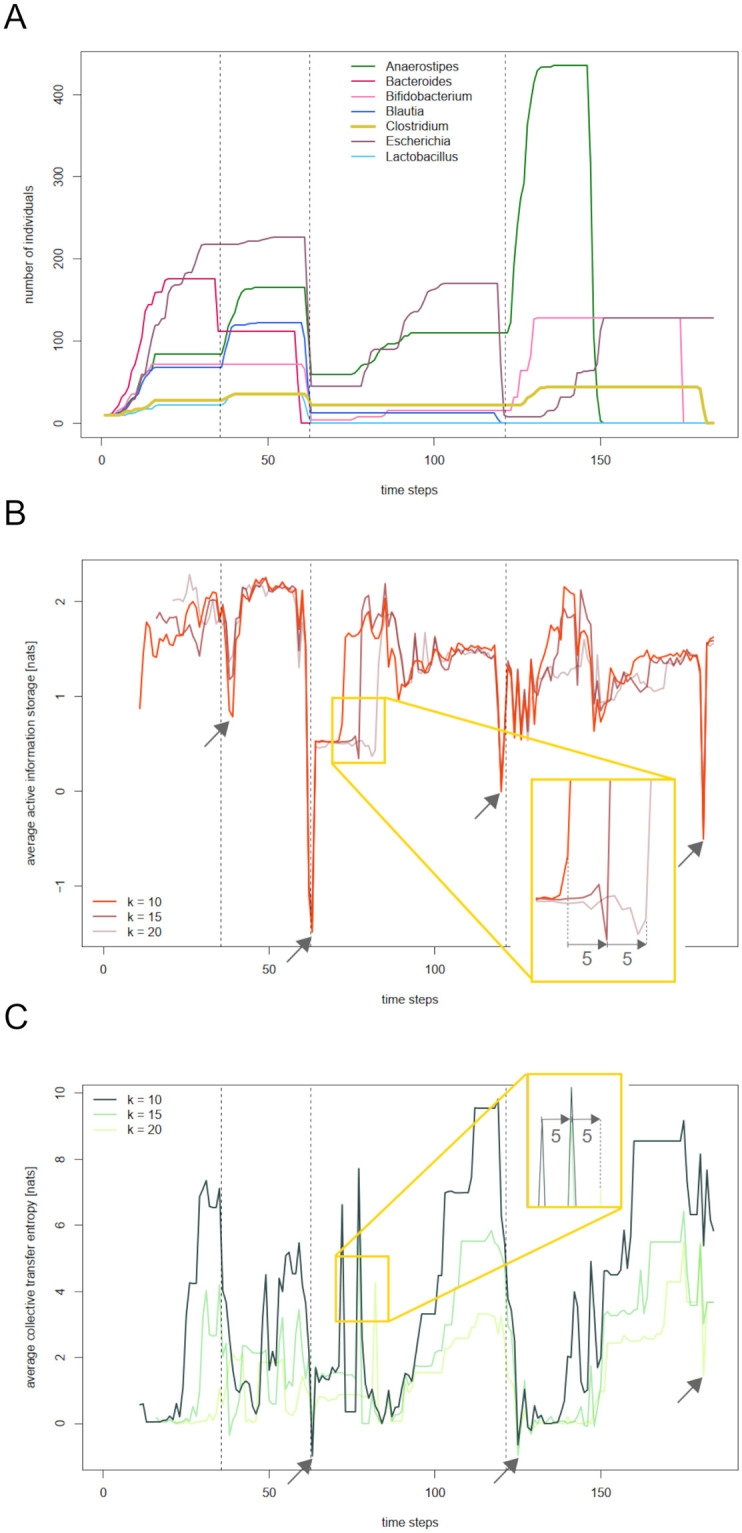
Information theoretic measures of the simulated SIHUMI community. A: Abundances of the seven SIHUMI species *Anaerostipes caccae, Bacteroides thetaiotaomicron, Bifidobacterium longum, Blautia producta, Clostridium ramosum, Escherichia coli, and Lactobacillus plantarum* in a run of the base simulation. Dashed vertical lines mark the feeding events after time steps 35, 62, and 121. B: Active information storage (AIS) averaged over all living species in the community for varying history lengths 10, 15, and 20. The yellow frame shows a pattern which is shifted by five time steps across history length. In contrast, the arrows point on noticeable simultaneous effects across history length. C: Collective transfer entropy (CTE) averaged over all living species in the simulated community for varying history lengths 10, 15, and 20.

First of all, we want to focus on the virtual abundance data, which will later serve as input for our information theoretical analysis. Fig 1A displays the abundances of the seven SIHUMI species in a single run of our BacArena base simulation. It is immediately apparent that the development of the abundances is strongly influenced by the feeding events, which are marked by dotted lines. The initial nutrient-rich environment leads to an increase in the number of individuals of all species. After some time, with essential nutrients becoming rare, the increase levels off, and *Bacteroides* even starts decreasing in abundance. This decrease is interrupted by the first feeding event after time step 35. The supply of nutrients leads to an increase in some species and prevents decrease in other species. After another 25 time steps, nutrient availability is getting critical again. Shortly before the second feeding event, all species strongly decrease in abundance, with *Bacteroides* and *Lactobacillus* even getting extinct. The nutrient refill after time step 62 avoids further decrease in the rest of the species and, after a couple of steps, some species start to build up abundance again. Following a period of constancy in all abundances, the pattern repeats, with *Blautia* getting extinct and *Escherichia* strongly decreasing in abundance. Nutrient supply interrupts this decrease and, again, leads to an increase in the abundances of the other species. Some time steps later, abundances level off, and, with no further nutrient input being provided, the species get extinct one after another. Only *Escherichia* is still present at the end of the simulation.

Based on the species’ abundance data, we can now derive the system’s information processing. Fig 1B displays the corresponding active information storage (AIS) averaged over all living species. Note that slight fluctuations in AIS during phases of constant abundances are a technical artefact due to the random noise added in the estimation process (compare [26]). Apparently, sudden large changes in community composition are always accompanied by downward ‘outliers’ in AIS across history length. The arrows in Fig 1B mark the most noticeable of those ‘outliers’. Around the second feeding and right before the end of the simulation, AIS even takes negative values, indicating that the past is misinformative of the current value of abundance (compare the Methods Section).

The collective transfer entropy (CTE) averaged over all living species is displayed in Fig 1C. Again, arrows mark simultaneous downward ‘outliers’, which take place at the second feeding event, after the third feeding event, and shortly before the end of the simulation. There is a gradual buildup of CTE before the feeding events, coinciding with periods of constant abundances across the community.

Considering CTE for varying history lengths 10, 15 and 20 (the *k-variants*), we observe that some patterns of the signals are shifted by exactly 5 time steps between history lengths 10 and 15, as well as between history lengths 15 and 20. Similar effects can be observed for AIS. Noticeable examples are highlighted by yellow frames in Fig 1B and 1C. This phenomenon results from a technical artefact, which will be discussed later.

### Information transfer reflects coherence in reaction to environmental changes

**Fig 2:**
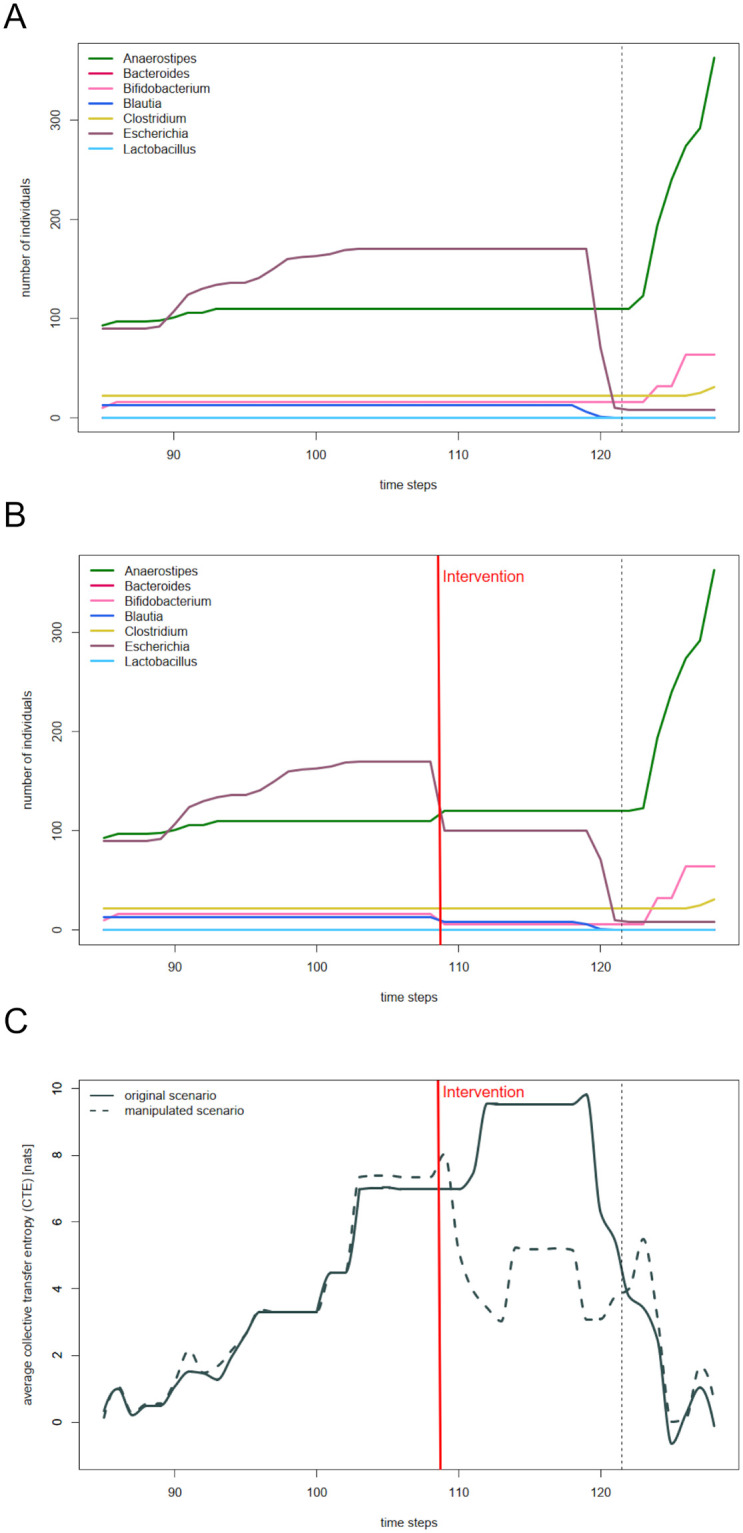
Disturbance of coherent behaviour leads to a decrease in information transfer. A: Abundances of the seven SIHUMI species between time steps 85 and 128 for a run of the base simulation. The dashed vertical line marks the feeding events after time step 121. B: Manipulated abundances of the seven SIHUMI species between time steps 85 and 128. C: Comparison of summarized abundances and collective transfer entropy averaged over all living species in the non-manipulated (solid lines) and manipulated (dashed lines) scenario.

If information transfer indeed captures coherent reactions to environmental changes, a perturbation of coherence should lead to a decrease in information transfer. We test this by manipulating results of the base simulation in a window between the second and third feeding (Fig 2A). Naturally, the further the second feeding event dates back, the lower the overall availability of essential nutrients. This gradual change in environmental conditions eventually leads to a collective stop in growth. With constant abundances in all species, the collective transfer entropy averaged over all living species becomes maximal during this time window (recall Fig 1C). We disturb the coherent behavior by randomly altering abundances between time steps 109 and 128 (Fig 2B).

Fig 2C displays the averaged collective transfer entropy of all living species both for the non-manipulated (solid lines) and the manipulated realization (dashed lines). In the non-manipulated realization, average CTE reaches its highest plateau of almost 10 nats at time step 112. At that time, abundances of all species have been constant for a while and continue to do so. In the manipulated realization, average CTE never reaches this level. With abundances changing at time step 109, average CTE slightly increases, followed by a decrease down to less than 3 nats. Throughout the next time steps, it fluctuates between this value and a maximum of just below 6 nats. It is only as of time step 125 that CTE in the two scenarios coincide again. Hence, by disturbing a community-wide coherent reaction to environmental changes, CTE can indeed be diminished (see S1 Fig for the k-variants of this signal).

### Information transfer reflects coherence resulting from effective interactions

**Fig 3:**
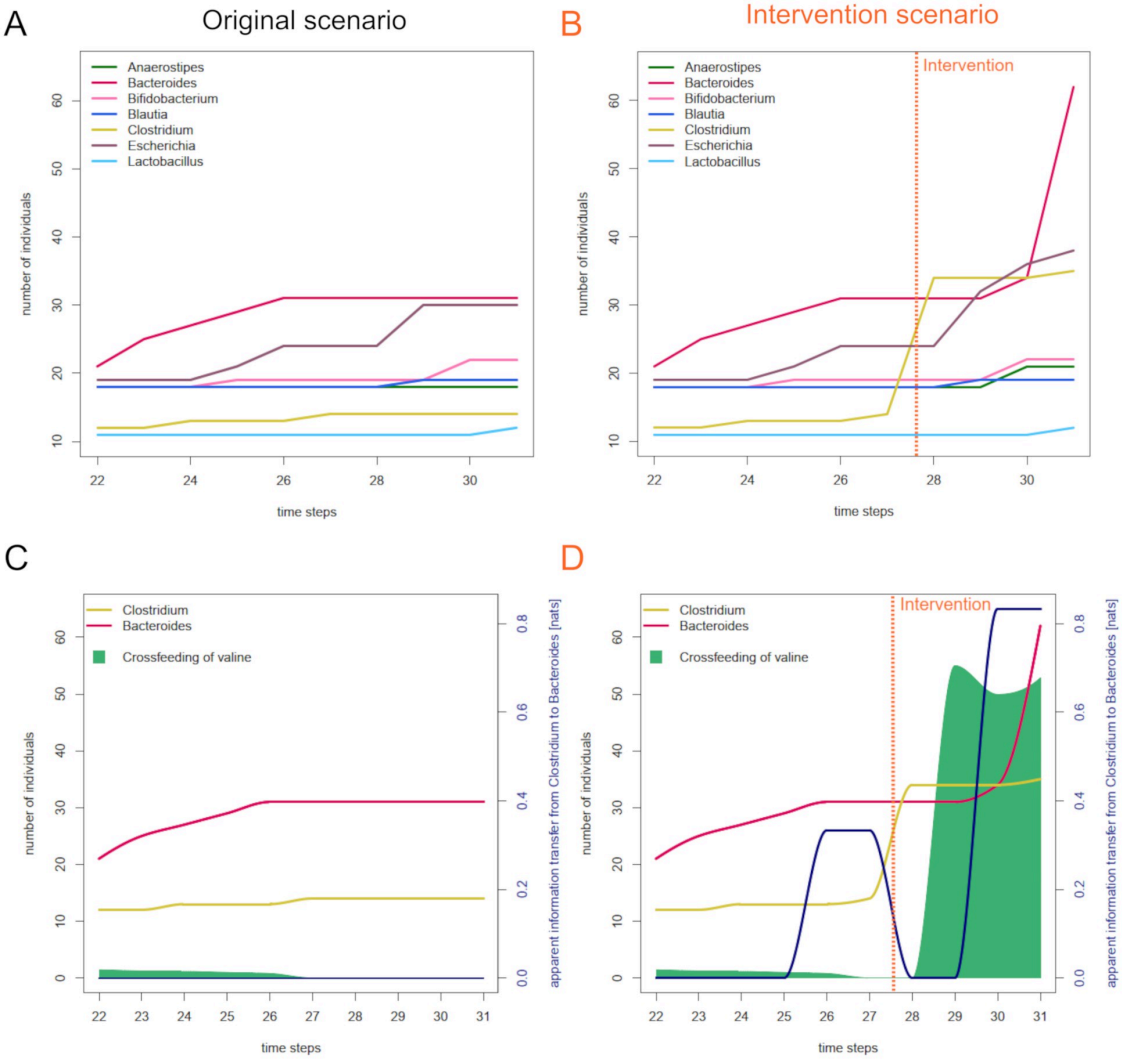
Direct interactions can be measured by information transfer. A: Abundances of the seven SIHUMI species between time steps 22 and 31 simulated in a 10x10 arena, starting off with ten individuals per species and a quarter of the base feeding. B: Abundances of the species in a second run, in which 20 individuals of *manipulated Clostridium* are introduced into the arena at time step 28. C: Comparison of crossfeeding of valine and apparent information transfer from Clostridium to Bacteroides in the non-manipulated scenario. D: Comparison of crossfeeding of valine and filtered apparent information transfer from Clostridium to Bacteroides in the manipulated scenario.

We test our second hypothesis by enforcing an effective direct interaction between two species. A resulting increase in the (apparent) information transfer between those two species would support our hypothesis. In preparation, we generate a new strain of *Clostridium*, whose reactions have been changed towards a larger production of L-valine. We intervene in a simulation by adding individuals of *manipulated Clostridium* to provoke a crossfeeding of L-valine, which has been shown to stimulate the growth of *Bacteroides* (compare S3 Fig). In Fig 3, both strains of *Clostridium* are grouped together under *Clostridium*.

Fig 3A and 3B display the abundances of the seven species between time steps 22 and 31 for a non-manipulated and a manipulated run of the simulation, respectively. In the non-manipulated case, *Clostridium* slightly increases in abundance between time step 26 and 27, *Bacteroides* remains constant as of time step 26. In the manipulated case, the addition of several individuals of *manipulated Clostridium* at time step 28 is followed by a slight increase in the abundance of *Bacteroides* at time step 30, leading into a subsequent steep increase. Fig 3C and 3D illustrate the contribution of L-valine to this growth: The amount of L-valine being produced by *Clostridium* and being consumed by *Bacteroides* increases significantly right after the addition of *manipulated Clostridium*, and right before the increase in abundance of *Bacteroides*. Hence, we have created a scenario in which crossfeeding leads to a change of abundances—an *effective interaction*. This effective interaction is indeed reflected in the corresponding transfer entropy, as a comparison between Fig 3C and 3D shows. While no information is transferred from *Clostridium* to *Bacteroides* in the non-manipulated cases (independent of history length *k*), transfer entropy takes values above 0.8 nats concurrently to *Bacteroides’* increase in abundance in the manipulated case (see S2 Fig for the k-variants of this signal).

Apart from the expected increase in transfer entropy at time step 30, we observe a lower plateau between time steps 26 and 27. This might surprise since abundances have not changed in this period and there is no signal in the non-manipulated scenario. However, we have to bear in mind that local transfer entropy is estimated on basis of the whole time series. Therefore, changing the time series at some point in time can lead to changes in local transfer entropy at other points in time.

## Discussion

### Interpreting information storage and transfer

Note that from an experimental perspective, the data resulting from our simulation appears exceptional to some extent. Longer periods of constant abundances are quite unlikely to be observed in natural systems due to various sources of uncertainty. However, following our reductionist approach using BacArena, the data still captures the essentials of bacterial community interaction we need to focus on. At the same time, the special structure of our data allowed us to identify artefacts of entropy estimation which would have been difficult to identify in analog estimates based on noisy observational data. However, filtering out such estimation artefacts is substantial for the interpretation of the information theoretical signals.

We have observed that active information storage is a mere reflection of sudden changes in the underlying abundances (recall Fig 1B). This relation between abundance data and AIS is not surprising. Active information storage quantifies the degree of predictability of a species’ abundance from its past –which is surely low whenever abundance suddenly increases or decreases after a period of constancy. The fact that AIS can serve as an indicator of changes in composition or behavior of a complex system has been discussed before, see e.g. [6] for experiments on swarm dynamics, or [13] for neural information processing. In our case, in which AIS of an agent is estimated on basis of time series of abundance data, only, it can be seen as a pure indicator of sudden changes in the underlying time series, and therefore cannot be expected to contain further information about the system.

In contrast, we have seen that (collective) information transfer captures “coherent” development in species’ abundances, both as a result of environmental changes and in reaction to crossfeeding. It can be assumed that further types of interaction, like competition over nutrients or the exposure to detrimental by-products of other species, may lead to an increased information transfer as well. In general, effective synergistic or competitive interactions are difficult to enforce in the SIHUMI community. The species in this community rather seem to coherently react to environmental changes. Indeed, there is growing evidence that the gut microbiome is shaped by habitat filtering rather than direct synergistic interactions (see e.g. [27, 28]), meaning that community assembly is dominated by the availability of nutrients rather than by the direct metabolic interaction among the bacteria being present. Our simulation experiments following realistic scenarios also support this claim. The main drivers of abundances and thereby also of the course of transfer entropy obviously are the feeding events. Coherent reactions to environmental changes follows the availability of nutrients. The sheer abundance of nutrients in combination with the mostly generalist metabolic repertoire of the chosen species seems to render competition less important. Furthermore, abundant resources being available appear to reduce the pressure on the bacteria to directly interact. One might hypothesize that this would change for a community in a rather restricted environment.

### The impact of history length in measuring information storage and transfer—Distinguishing immediate and delayed effects

In order to understand the origin of the “shifted patterns” observable in the information theoretic signals across history lengths (Fig 1B and 1C), compare the definition of active information storage for history length *k* at time step *x*_*n*+1_ (Eq (3), below). One conditions on a certain past state *x*_*t*_ in the estimation of AIS at time steps *x*_*t*+1_, …, *x*_*t*+*k*_. The same holds for collective transfer entropy (Eq (5), below). Therefore, if increasing the history length by *s* leads to a shift of some feature in AIS/CTE from *x*_*n*+1_ to *x*_*n*+1+*s*_, this suggests that the observed feature is related to omitting *x*_*n*−*k*_ from the conditioned past rather than to the transition from *x*_*n*_ to *x*_*n*+1_. We call such a feature a *delayed effect*, since it reflects a past development. Unrecognized delayed effect bear the risk of strong misinterpretations. In contrast, if some feature in AIS/CTE is temporally stable at *x*_*n*+1_ across history lengths, we may confidently relate it to the development in abundances between *x*_*n*_ and *x*_*n*+1_. We call such a feature an *immediate effect*.

**Fig 4:**
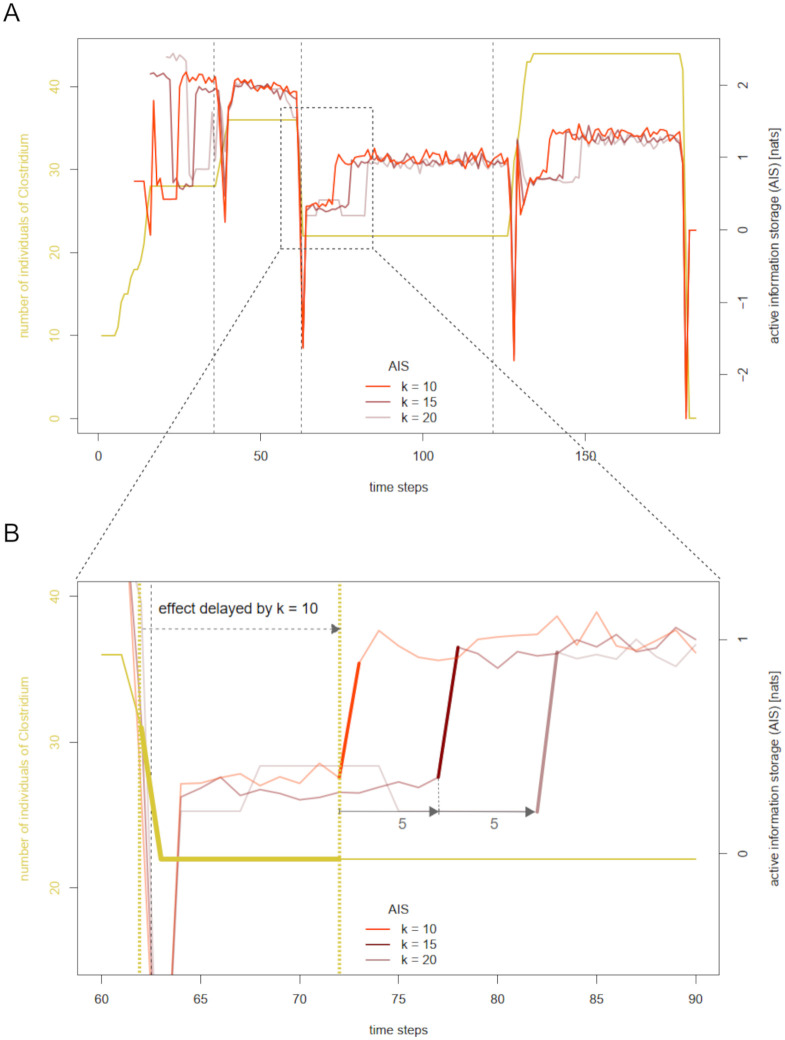
Identifying immediate and delayed effects in the active information storage of *Clostridium*. A: Abundance and active information storage (AIS) of *Clostridium* with varying history lengths 10, 15, and 20 for a run of the base simulation. Dashed vertical lines mark the feeding events after time steps 35, 62, and 121. B: Illustration of a delayed effect in the AIS of *Clostridium*. The highlighted sections of the k-variants of AIS are shifted by exactly five time points, indicating that they capture an effect lagging behind by the respective history length.

**Fig 5:**
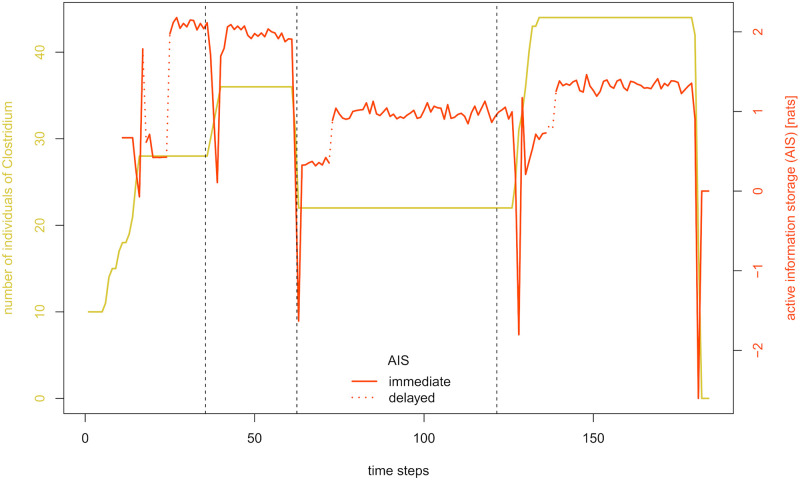
Filtered active information storage of *Clostridium*. Filtered active information storage (AIS) of Clostridium for a run of the base simulation with delayed effects marked by dotted lines. Dashed vertical lines mark the feeding events after time steps 35, 62, and 121.

We will further illustrate this phenomenon in the information theoretical signals of *Clostridium*. Fig 4A displays its abundance and active information storage for history lengths 10, 15, and 20. Fig 4B allows a closer look at an apparent shifted pattern after the third feeding event. The strong increase in AIS for *k* = 10 between time steps 72 and 73 repeats itself five time steps later for *k* = 15 and ten time steps later for *k* = 20. This hints towards the fact that the cause for this pattern in AIS is not *Clostridium’s* abundance between time steps 72 and 73 (nor its abundance between time steps 77 and 78 or time steps 82 and 83, respectively). Instead, we have to relate this pattern to the omission of time step 62 from the conditioned past. In this case, the shift of the conditioned past onto the plateau of constant abundance naturally increases the informative value of the past on the next value of abundance. Following this procedure, we identify all shifted patterns in the signal. Fig 5 illustrates the result of our identification process, with dotted lines indicating the periods where a clear identification of the signal is difficult due to superposition of shifted and non-shifted features. As expected, the strong downward outliers are all immediate effects, being clearly related to the sudden increases or decreases in abundance. In contrast, several increases of AIS are delayed effects, which are not primarily related to the concurrent development of abundance, but to the fact that the conditioned past is being successively shifted on plateaus of constant abundance.

**Fig 6:**
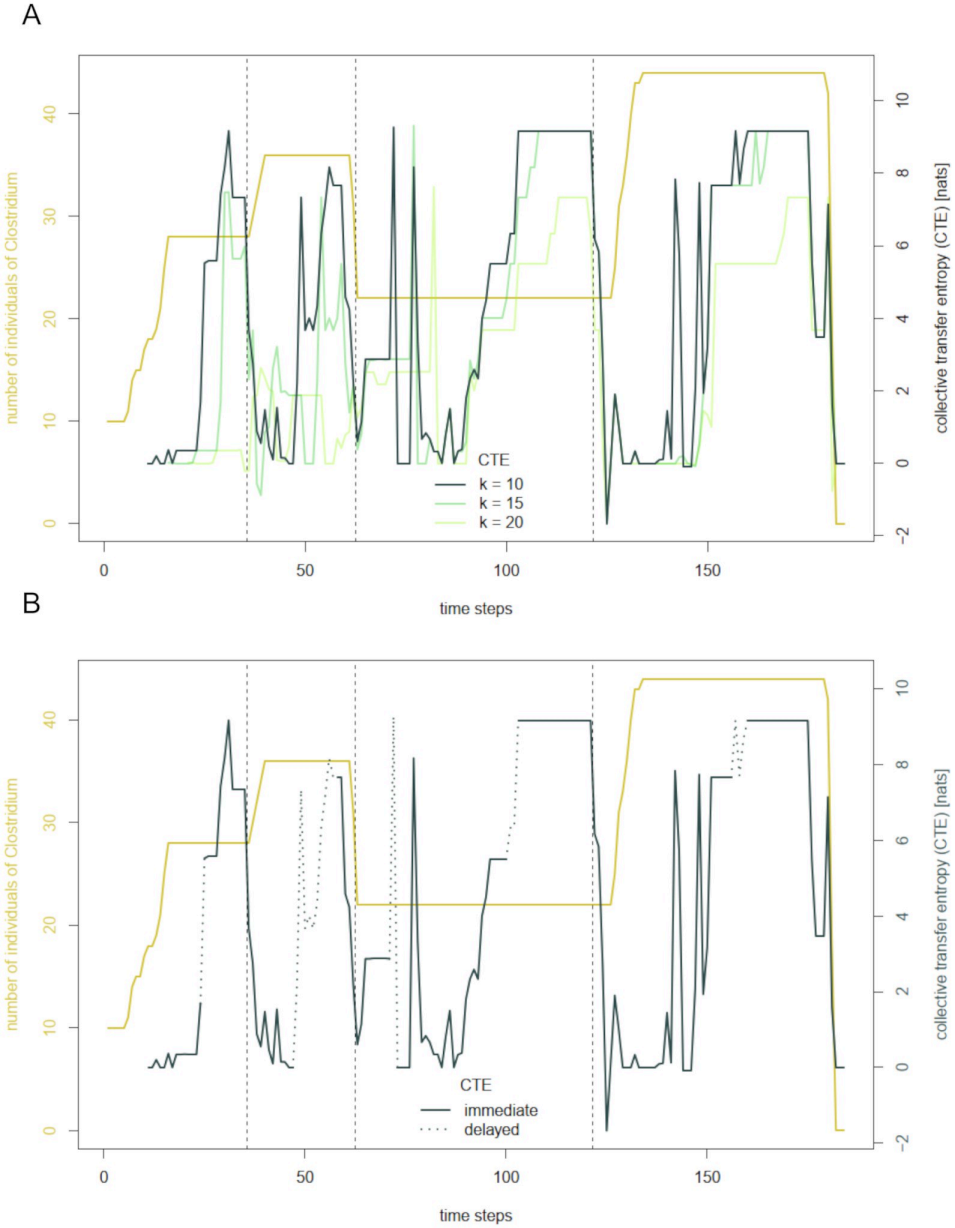
Filtering the collective transfer entropy of *Clostridium*. A: Abundance and collective transfer entropy (CTE) of *Clostridium* with varying history lengths 10, 15, and 20 for a run of the base simulation. Dashed vertical lines mark the feeding events after time steps 35, 62, and 121. B: Filtered collective transfer entropy (CTE) of Clostridium with delayed effects marked by dotted lines.

The procedure is analog in the case of collective transfer entropy. Fig 6A displays the abundance and collective transfer entropy of *Clostridium* for history lengths 10, 15, and 20. As already implied by the average CTE in Fig 1C, CTE tends to build up during periods of longer constancy. From time to time, the signal shows strong upward and downward outliers. Again, indicating ambiguous parts by dashed lines results in a “filtered” version of the CTE of *Clostridium* (see Fig 6B). As we have already seen for AIS, it is mainly increases of CTE during times of constant abundance, which are identified as delayed effects.

### The relation of information storage and transfer

**Fig 7:**
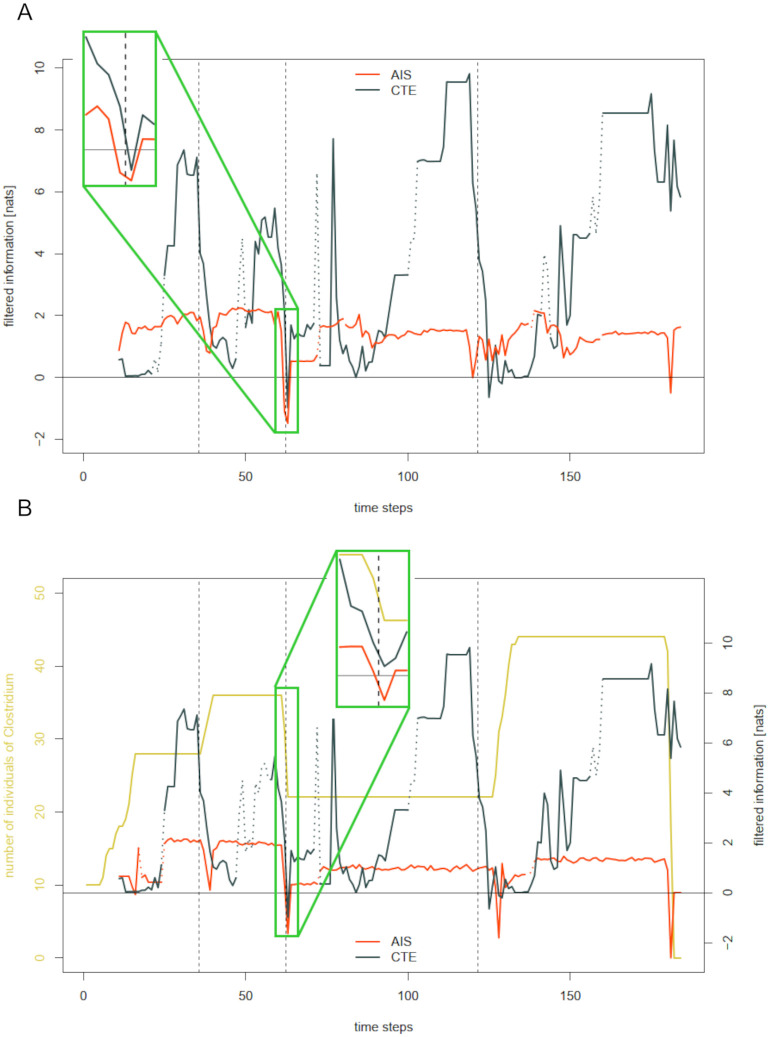
Comparing active information storage and collective transfer entropy. A: Filtered active information storage (AIS) and collective transfer entropy (CTE) for a run of the base simulation averaged over all living species in the SIHUMI community. Dashed vertical lines mark the feeding events after time steps 35, 62, and 121. B: Filtered active information storage (AIS) and collective transfer entropy (CTE) of *Clostridium*.

Wang et al. [6] studied collective communication and memory in the spatiotemporal dynamics of simulated swarms, suggesting general principles for distributed computation in social and biological systems. The authors observe that average maximal information transfer tends to follow maximal information storage. We observe this pattern as well in the sense that with compositional changes around feeding events, which lead to declines in both information theoretic measures, AIS tends to build up and reach its maximal plateau quicker than CTE (compare Fig 7A and 7B for community-wide and species-specific signals). This phenomenon can easily be explained: In our simulation, a drop in AIS of a species is caused by either a sudden increase in abundance (after a feeding event) or a sudden decrease in abundance (with essential nutrients missing). Those disturbances are followed by either constant abundance (which renders the next state of abundance highly predictable from its past), or by a steady increase (which is, as well, a stable and thereby predictable trend). In contrast, high CTE requires uniformity in preferably many species or effective direct interactions among them. Both is not given during the phases of disturbance. Species react differently, both with respect to shortage of nutrients, as well as to the subsequent feeding events. However, it is not interactions driving their abundances. Only after abundances have been simultaneously constant over some period, CTE reaches maximal values.

Additionally, the results presented in [6] suggest that information transfer alternates with information storage. While this may be valid for a wide range of disturbed complex systems, it does not hold true in our specific scenario. Recall that an individual in the swarm simulated in [6] has effectively two possible sources of information: it’s own past, which dominates in phases of “coordinated” collective behavior, and other individuals which become more important in phases of disturbance. However, in our setting there is a third crucial source of information which is the system’s environment. One could think of the environment as constituting an additional agent governing the availability of nutrients. Lack of, or a sudden refill of nutrients clearly shapes this other agent’s “behavior”. Neither AIS nor CTE will be able to capture such interaction directly since the environment is not considered as an agent in the system. And indeed, both measures drop after the feeding event (compare Fig 7A and 7B), indicating the past to be misinformative about the future. In such times of disturbance, the only measure that we would expect to increase is intrinsic uncertainty (compare the Methods Section).

Nevertheless, we have seen earlier that there are situations in which species’ reactions to the environment lead to an increase in (collective) transfer entropy. It will be an interesting future task to find ways to distinguish environmentally-induced from interaction-driven transfer signals.

## Conclusion

We took first steps into the study of the human gut microbiome as computing entity. First steps only, since we restricted ourselves to a *simplified* and *simulated* community. That allowed us to directly intervene in the system to confirm two hypotheses: Information transfer among the species reflects coherent behavior both as a reaction to environmental changes and in form of direct effective interactions. In particular, the latter will serve as a valuable tool when being applied to real data. It could provide insight into little understood or so far unknown relationships and dependencies between bacterial species. Such knowledge can be crucial when it comes to the design of dietary measures or medical intervention strategies. Hence, the transfer of our work to real data provides a natural next step. Albeit rare, suitable long-term time series of the composition of the human gut microbiome do exist.

Using a rather constrained simulation for the basis of our analysis allowed us to decipher the artefacts of entropy estimation which must be taken into account when interpreting courses of local AIS and CTE. Currently, we chose to manually distinguish immediate and delayed effects in the signals. However, for wider applicability, an automatized process would be desirable. Such a process might for example be developed via objective decision criteria based on *k*-variants of the signals.

## Materials and methods

A suitable experimental playground for our research questions is provided by BacArena, a software for the simulation of microbial communities, combining agent-based modelling and flux balance analysis [25]. The time series of abundance data resulting from these experiments serve as basis for our information theoretical analysis of the underlying system.

### Simulating a microbiome with BacArena

The experimental setup of our base simulation appears as case study of an integrated multi-species model of the human gut in [25]. The model was assembled using manually curated genome-scale metabolic models of the following seven human gut bacteria [29]:

- *Anaerostipes caccae DSM 14662*
- *Bacteroides thetaiotaomicron VPI 5482*
- *Blautia producta DSM 2950*
- *Escherichia coli str. K 12 substr. MG1655*
- *Clostridium ramosum VPI 0427 DSM1402*
- *Lactobacillus plantarum subsp. plantarum ATCC 14917*
- *Bifidobacterium longum NCC2705*

The models, as being used in [25], are provided in the supplementary material (see S1 Data). This community, which also has been used in *in vitro* and in animal experiments, comprises representatives of the functionally most relevant groups of the human gut microbiome. As such, it has been established as a simplified human intestinal microbiota (SIHUMI) [23].

Using the R-version of BacArena, we set up a 100 x 100 grid, the so-called *arena*. Following the set-up by the authors of BacArena [25], the arena was initialized with all nutrients which can be consumed by at least one species expect mucus glycans. Per default, all nutrients were set to a concentration of 0.1 μM per cell. Based on flux variability analysis [30], the nutrients essential for a species-wise biomass growth rate of 0.01h^−1^ were determined. In order to ensure this growth, the concentration of the corresponding *essential* nutrients was increased to 1 μM per grid cell. Overall, the medium consists of 269 nutrients, 29 of which are classified as essential. The exact diet definition can be found in S1 Table. We will denote this composition as *base feed/medium*. 10 individuals of each species were randomly placed on the grid. Default values were used for growth and movement parameters (compare [25], Table 3). After time steps 35, 62, and 121, the initial amount of nutrients was added to the medium again (*feeding events*). After the third feeding event, the simulation was continued until only one species was alive, leading to a simulation length of 184 steps. The R code underlying this simulation is provided in S1 Code. Large parts of our analysis are based on the species abundances resulting from one run of the base simulation. These time series can be found in S2 Table.

In order to test our hypotheses on the biological interpretation of transfer entropy, we perform two interventions. According to our first hypothesis, transfer entropy captures coherent behavior in the form of a coherent reaction to environmental changes. Hence, we disturb a period of formerly constant abundances in the base time series by randomly increasing or decreasing abundances following time step 109. The manipulated time series data is provided in S3 Table.

According to our second hypothesis, transfer entropy reflects direct effective interactions between species. To test this hypothesis, we enforce such an interaction between two species. In preparation, we conducted simulations to identify products of one SIHUMI species whose availability leads to a strong growth of another SIHUMI species. In doing so, we learned that the combined increased availability of phosphate and the amino acid L-valine leads to a growth in abundance of *Bacteroides* (compare S3 Fig). Among the SIHUMI species, one of the main producers of L-valine is *Clostridium*. However, the regular amounts produced are too small to effectively lead to significant changes in abundance of *Bacteroides*. Therefore, we change the corresponding reaction in the model of *Clostridium* towards 1000-fold output of L-valine. In the following, we will refer to this new strain as *manipulated Clostridium*. In order to increase controllability, we set up a new simulation on a smaller grid (30 x 30) with randomly distributing ten individuals of each of the seven SIHUMI species. We use a quarter of the base feeding as initial medium.

We run the simulation until 38 time steps are reached. The resulting time series of abundance data can be found in S4 Table. In a second run, we intervene at time step 28. Specifically, we add 20 individuals of manipulated *Clostridium*, 6.25 fmol per cell of glutamate, which *Clostridium* needs to produce L-valine, and 6.25 fmol per cell of phosphate to the medium and continue the simulation for another 10 time steps. The source code underlying the simulation is provided in S2 Code. The resulting abundance data can be found in S4 Table.

### Information decomposition

Our two operations of interest, information storage and transfer, are ultimately related by the theory of information decomposition (see e.g. [7]). In the following, we define the two operations in this context.

Let V={…,X,Y,Z,…} be a system of countably many stationary discrete Markov processes, or, as we will call them in the following, *agents*. Let *p*(*x*) denote *p*(*X* = *x*), *p*(*y*) = *p*(*Y* = *y*), etc. The information being contained in a state *x*_*n*+ 1_ of an agent *X* can be quantified by the agent’s *(local) entropy*

$$\begin{array}{c} h_{X}\left(x_{n+1}\right)=-\text{log}(p(x_{n+1})). \end{array}$$ (1)

In other words, *h*_*X*_(*x*_*n*+1_) quantifies the amount of uncertainty captured in the measurement *x*_*n*+1_ at time point *n*. One can distinguish three possible sources for this information.

The first source is the agent’s own past. Let *k* denote the agent’s Markov order, i.e.

$$\begin{array}{c} p\left(x_{i+1}|x_{i},\ldots ,x_{i-k+1}\right)=p\left(x_{i+1}|x_{i},\ldots ,x_{i-k+1},x_{i-k}\right) \end{array}$$ (2)

In the following, we will use the notation $x_{i}^{\left(k\right)}=\left(x_{i},\ldots ,x_{i-k+1}\right)$. Then, the *local active information storage AIS* [7] quantifies the amount of information in *x*_*n*+1_ which is predictable from the past state $x_{n}^{\left(k\right)}$, i.e.

$$\begin{array}{c} a_{X}\left(x_{n+1},x_{n}^{\left(k\right)}\right)=\text{log}(\frac{p(x_{n+1}|x_{n}^{\left(k\right)})}{p(x_{n+1})}). \end{array}$$ (3)

Note that local AIS is negative whenever

$$\begin{array}{c} p(x_{n+1}\;|\;x_{n}^{\left(k\right)})<p\left(x_{n+1}\right). \end{array}$$ (4)

In this case, $a_{X}\left(x_{n+1},x_{n}^{\left(k\right)}\right)$ measures the amount of misinformation about *x*_*n*+1_ by $x_{n}^{\left(k\right)}$.

The second source consists of information provided by other agents and not being contained in *X*’s own past. Let $V_{X}\subset V\backslash X$ be the subset of all possible information contributors of *X*. This set can be determined on basis of background knowledge of the system or using computational methods [10]. Let ${\vec{v}}_{X,n}^{\left(l\right)}$ be the vector of states of the agents in $V_{X}$ obtained by concatenation, with **l** being the vector of their respective Markov orders. Information being predictable from the past of other agents but not from the past of *X* itself is measured by the *(local) collective transfer entropy CTE* [7]

$$\begin{array}{c} t_{X}\left(x_{n+1},x_{n}^{\left(k\right)},{\vec{v}}_{X,n}^{\left(l\right)}\right)=\text{log}(\frac{p(x_{n+1}\;|\;x_{n}^{\left(k\right)},{\vec{v}}_{X,n}^{\left(l\right)})}{p(x_{n+1}\;|\;x_{n}^{\left(k\right)})}). \end{array}$$ (5)

This measure includes interaction-based as well as single-sourced transfers to *X*.

To infer the contribution of a single agent on *X*, we can restrict V to one element. Indeed, let $Y\in V_{X}$ be an agent with Markov order *l*. Then, *(local) apparent transfer entropy TE* [16] from *Y* to *X* is defined as

$$\begin{array}{c} t_{Y\to X}\left(x_{n+1},x_{n}^{\left(k\right)},y_{n}^{\left(l\right)}\right)=\text{log}(\frac{p(x_{n+1}\;|\;x_{n}^{\left(k\right)},y_{n}^{\left(l\right)})}{p(x_{n+1}\;|\;x_{n}^{\left(k\right)})}). \end{array}$$ (6)

Transfer entropy can be understood as the average amount of information in the source *Y* about the next state of the destination *X* that was not already contained in the past of *X* itself. Note that the collective transfer entropy is not the sum of the apparent transfer entropies from all sources but a sum of incrementally conditioned transfer entropies [7]. Analogously to the case of local AIS, negative local (collective) TE indicates that the sources’ past states have been misinformative about the agent’s next state. An expected causal *delay* between source and destination variable can be taken into account by replacing $y_{n}^{\left(l\right)}$ by $y_{n+1-d}^{\left(l\right)}$ and ${\vec{v}}_{X,n}^{\left(l\right)}$ by ${\vec{v}}_{X,n+1-d}^{\left(l\right)}$.

The third source comprises all information which is neither stored in the agent’s past nor being transferred from other agents. It is called *local intrinsic uncertainty U* and consequently measured by [7]

$$\begin{array}{c} u_{X}\left(x_{n+1},x_{n}^{\left(k\right)},{\vec{v}}_{X,n}^{\left(l\right)}\right)=\text{log}(p(x_{n+1}\;|\;x_{n}^{\left(k\right)},{\vec{v}}_{X,n}^{\left(l\right)})). \end{array}$$ (7)

Hence, the information needed to predict the next state of an agent is a composition of information being stored in the agent’s own past, information being transferred from other agents, and intrinsic uncertainty. Formally, this can be expressed as [7]

$$\begin{array}{c} h_{X}\left(x_{n+1}\right)=a_{X}\left(x_{n+1},x_{n}^{\left(k\right)}\right)+t_{X}\left(x_{n+1},x_{n}^{\left(k\right)},{\vec{v}}_{X,n}^{\left(l\right)}\right)+u_{X}\left(x_{n+1},x_{n}^{\left(k\right)},{\vec{v}}_{X,n}^{\left(l\right)}\right). \end{array}$$ (8)

### Implementation

We consider the seven species of the simulated SIHUMI community as agents of a complex system V. We identify each of them with a stationary discrete Markov process *X*. A time series of abundance data $\tilde{X}$ resulting from a simulation run can then be thought of as a specific realization of this Markov process. We consider all species as possible mutual information contributors due to competition over essential nutrients and space. Hence, we set $V_{X}=V\backslash X$ for every *X*.

In order to apply the information theoretic measures presented above, we need to choose two defining parameters. We set a delay of *d* = 2 in the transfer between species, since the change in abundance of a species will lead to a change in metabolic activity of another species at earliest in the next time step. Any change in abundance of another species will therefore occur at earliest in the next but one time step.

A species’ Markov order, or “informing past”, can often not be known but only be estimated. Apart from already implemented estimation methods, like the Ragwitz optimization provided with the JIDT toolkit [31], one can get hints on the past dependency structure by considering the time series’ active information storage for varying history lengths (compare e.g. [11]). Typically, average information storage of an agent increases with increasing history length *k* until *k* reaches the length of the agent’s intrinsic memory, where it tends to levels off or decline. However, the specific structure of our abundance curves provokes a different pattern. As Fig 1A displays, the abundances in our base simulation are characterized by constant periods, being interrupted by comparably short phases of increase or decrease. Clearly, an agent *X*’s active information storage at some time point *n* + 1 is relatively high if $\left({\tilde{x}}_{n}^{\left(k\right)},{\tilde{x}}_{n+1}\right)$ is constant or shows a uniform trend. However, the longer the history length *k*, the rarer this situation occurs. With this phenomenon being the dominant driver of AIS in our time series, average AIS tends to decrease with increasing history length.

**Fig 8:**
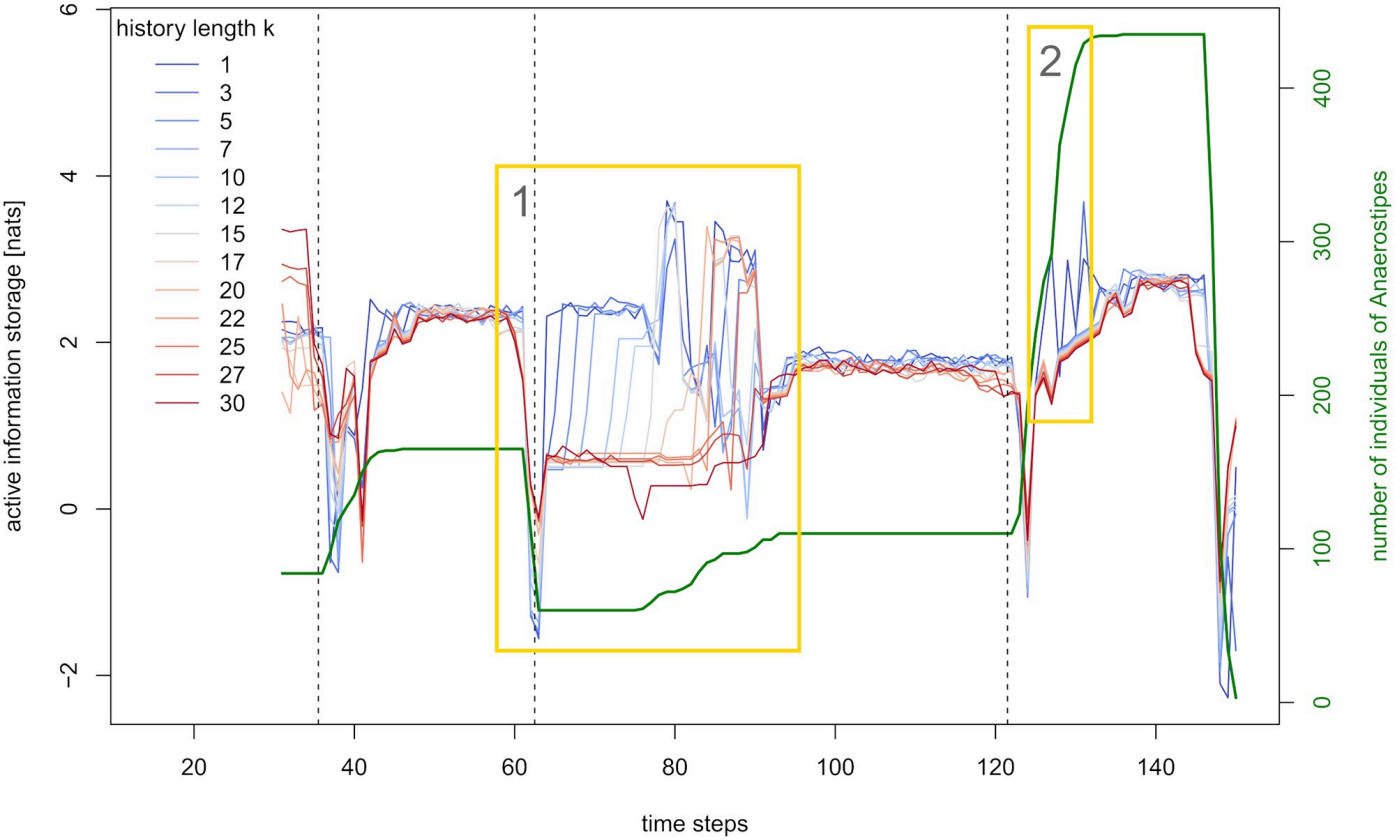
The effects of varying history length on active information storage of *Anaerostipes*. Abundance and active information storage of *Anaerostipes* for a run of the base simulation with history length *k* varying between 1 and 30. Dashed vertical lines mark the feeding events after time steps 35, 62, and 121. The yellow frames emphasize two types of differences across history lengths.

Fig 8 illustrates the phenomenon for the example of *Anaerostipes*, with history lengths varying between *k* = 1 and *k* = 30. We want to highlight two patterns, during which the differences between the history lengths become very clear. In frame 1 (see yellow box in Fig 8), the effect of the plateau of constant abundance on AIS decreases with increasing history length. While the existence of such a plateau could be easily derived from AIS curves with history length up to 20, the effect is growingly blurred for larger history lengths. Frame 2 displays extreme outliers for very small history lengths, capturing short periods of comparatively small slope in abundance. Hence, the choice of history length apparently is a balancing act between fine scanning, increasing susceptibility to noise, and coarse scanning, bearing the risk of averaging out signals relevant for the analysis.

The scope of our analysis is an insight into the behavior and, consequently, a refined application of information storage and transfer in the context of microbial communities. Fig 8 illustrates that both the restriction to only one history length as well as to extremely small or large history lengths is not adequate for this task. Therefore, we consider intermediate-sized history lengths of *k* = 10, 15 and 20 (and *l* = 9, 14 and 19, taking the delay of *d* = 2 into account) throughout this analysis. We will further discuss the observed phenomena in the Results Section.

The (joint) probabilities appearing in the formulas above have to be estimated. To this end, we make use of the R package QtAC (Quantifying the Adaptive Cycle) [26], which is available via https://github.com/hannahschrenk/QtAC. The package borrows the Kraskov-Stögbauer-Grassberger (KSG) estimator of the JIDT toolkit, an advanced kernel estimator being optimized to deal with small sample sizes [31]. By default it yields results in nats (natural units of information). QtAC contains estimators for local and average transfer entropy, collective transfer entropy, as well as active information storage. The (joint) probabilities are estimated based on the whole time series of (joint) abundances. Note that all information theoretic measures being displayed in this work are local measures. As of now, we will omit the addendum “local” to increase readability.

The information theoretic values being shown are averaged over 25 estimation runs. Based on the significance test included in QtAC, we only took results with a significance ≤ 0.05 into account. All parameter choices are listed in S5 Table.

## Supporting information

S1 Fig Collective transfer entropy under varying history lengths. Collective transfer entropy averaged over all living species in the SIHUMI community between time steps 85 and 128 for varying history lengths 10, 15, and 20 in the non-manipulated (A) and manipulated scenario (B).
(TIF)

S2 Fig Transfer entropy under varying history lengths. Information transfer from *Clostridium* to *Bacteroides* between time steps 85 and 128 for varying history lengths 10, 15, and 20 in the non-manipulated (A) and manipulated scenario (B).
(TIF)

S3 Fig The combined addition of L-valine and phosphate stimulates the growth of *Bacteroides*. Growth of *Bacteroides* in a 30x30 arena on the base medium (A) minus L-valine and phosphate, (B) minus phosphate but with an increased value of phosphate (6.25 fmol/cell), (C) minus phosphate but with an increased value of L-valine (6.25 fmol/cell), (D) with increased values of phosphate and L-valine (6.25 fmol/cell each).
(TIFF)

S1 Data Models of the seven SIHUMI species used in the base simulation. (RDATA)

S1 Table Nutrients added to the media at feeding events in the base simulation. (CSV)

S2 Table Time series of abundance data resulting from the base simulation. (CSV)

S3 Table Manipulated time series of abundance data of the base simulation. (CSV)

S4 Table Time series of abundance data resulting from the simulation with and without enforced interaction. (XLSX)

S5 Table Parameters used in the information theoretic computations with QtAC. (PDF)

S1 Code Source code of the base simulation. (RMD)

S2 Code Source code of the simulation with enforced interaction. (RMD)
