# Supplementary figures and images for: Measuring and understanding information storage and transfer in a simulated human gut microbiome
Source: PLoS Comput Biol. 2024 Sep 17;20(9):e1012359. doi: 10.1371/journal.pcbi.1012359 (PMC11407623; doi:10.1371/journal.pcbi.1012359)

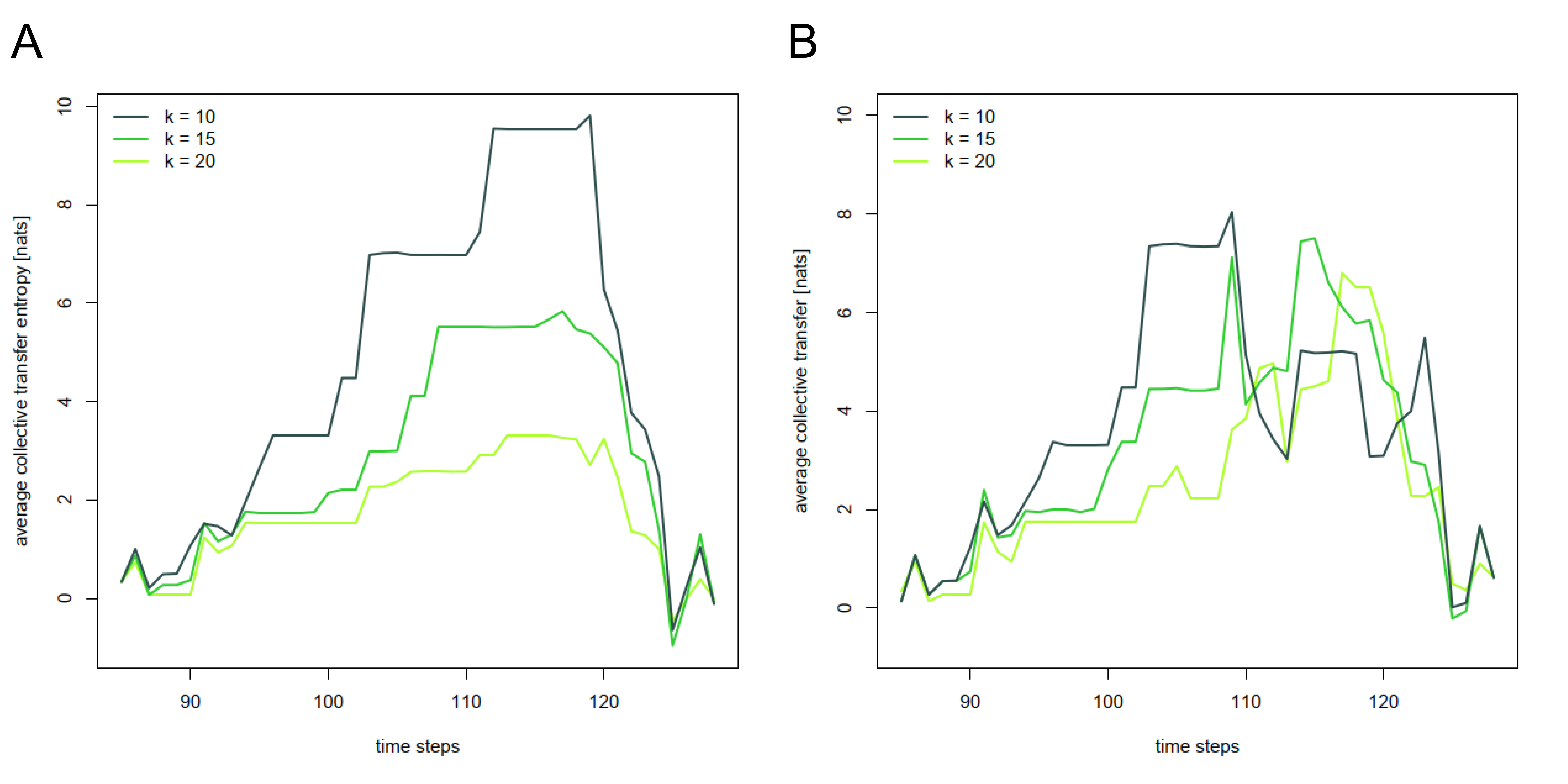

Supplement: S1 Fig — Collective transfer entropy averaged over all living species in the SIHUMI community between time steps 85 and 128 for varying history lengths 10, 15, and 20 in the non-manipulated (A) and manipulated scenario (B). (TIF) [file pcbi.1012359.s001.tif]

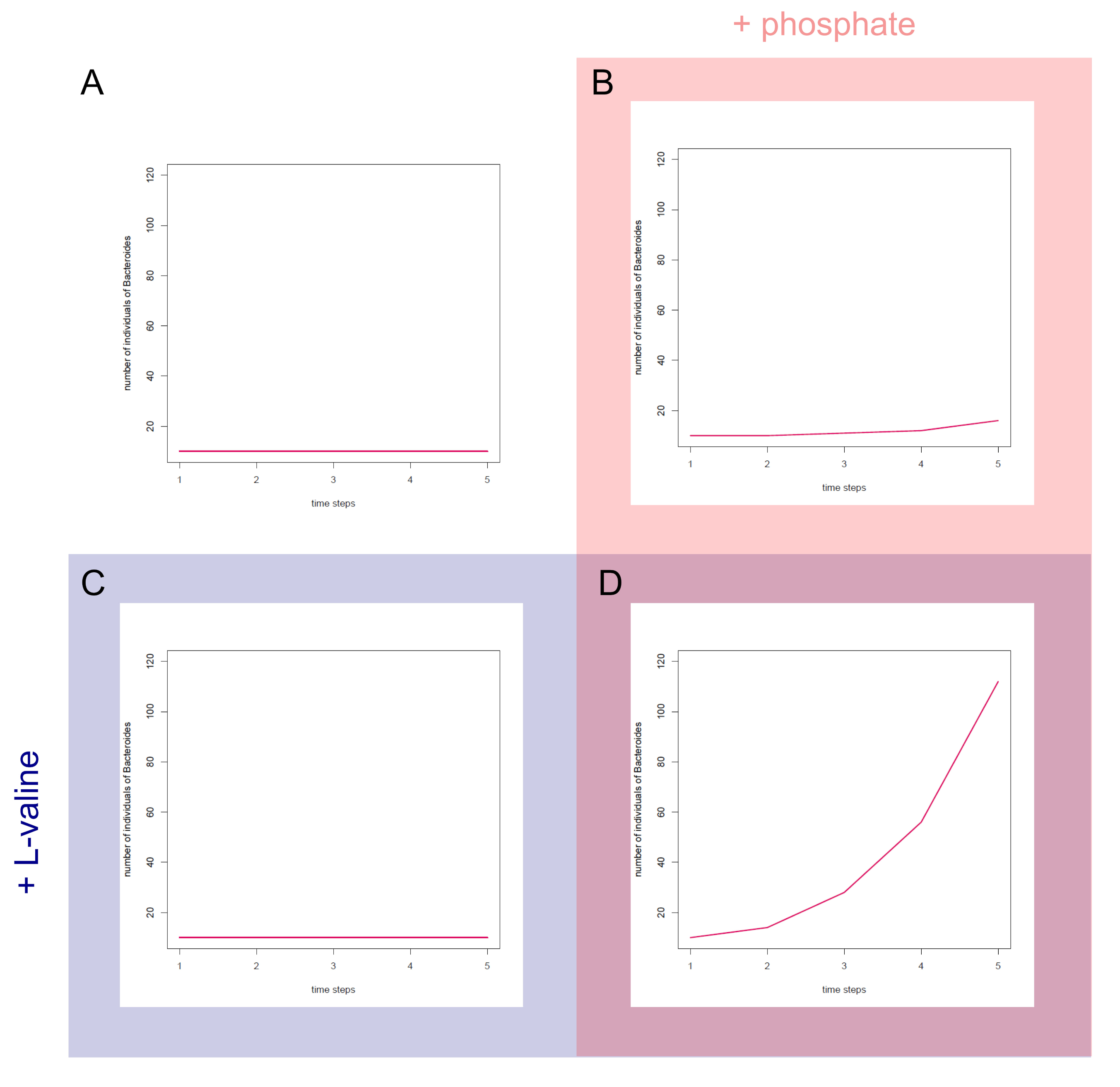

Supplement: S3 Fig — Growth of Bacteroides in a 30x30 arena on the base medium (A) minus L-valine and phosphate, (B) minus phosphate but with an increased value of phosphate (6.25 fmol/cell), (C) minus phosphate but with an increased value of L-valine (6.25 fmol/cell), (D) with increased values of phosphate and L-valine (6.25 fmol/cell each). (TIFF) [file pcbi.1012359.s003.tiff]
