# Supplementary material for: Measuring and understanding information storage and transfer in a simulated human gut microbiome
Source: PLoS Comput Biol. 2024 Sep 17;20(9):e1012359. doi: 10.1371/journal.pcbi.1012359 (PMC11407623; doi:10.1371/journal.pcbi.1012359)
Supplement: S5 Table — (PDF) [file pcbi.1012359.s009.pdf]

Parameter choices in QtAC

	QtAC_AIS	QtAC/QtAC_CTE
**mode**	"local"	"local"
**num_timepoints**	184	184
**k**	10	10
**l**		9
**k_tau**	1	1
**l_tau**		1
**delay**		2
**noise_level**	1.00E-08	1.00E-20
**num_permcheck**	1.00E+03	1.00E+03
**signfac**	0.05	0.05
